# ERBB signaling in CTCs of ovarian cancer and glioblastoma

**DOI:** 10.18632/genesandcancer.162

**Published:** 2017-11

**Authors:** Anjali Geethadevi, Deepak Parashar, Erin Bishop, Sunila Pradeep, Pradeep Chaluvally-Raghavan

**Affiliations:** ^1^ Department of Obstetrics and Gynecology, Medical College of Wisconsin, Milwaukee, Wisconsin, USA; ^2^ Department of Physiology, Medical College of Wisconsin, Milwaukee, Wisconsin, USA

**Keywords:** circulating tumor cells, ERBB receptors, ovarian cancer, glioblastoma

## Abstract

Circulating Tumor Cells (CTCs) are floating cell populations, which are resistant to anoikis after detachment from the primary sites and travel through the circulatory and lymphatic systems to disseminate throughout the body. CTCs are considered as seed cells for metastasis, and thus isolation of CTCs does not require any invasive procedure. Based on the nature and location of ovarian cancer and glioblastoma, the role of CTCs and hematogenous (carried by blood) spreading of tumor cells in these cancers were not understood well. Dysregulation of epidermal growth factor receptor (EGFR/ERBB) family members due to their overexpression and/or mutation have been known to contribute to the etiology and progression of ovarian cancer and glioblastoma. However, the role of ERBB receptors on CTC formation of ovarian cancer and glioblastoma is not well established. This report highlights the role of ERBB family receptors on resistance to anoikis and CTC formation in ovarian cancer and glioblastoma. Recent research on CTCs demonstrates that capturing ERBB receptor positive cells from circulating system is an efficient approach to isolate CTCs for genomic and proteomic characterization of tumor cells. Therefore, ERBB-targeted isolation of CTCs would help to design therapy to treat cancer, determine drug responses and drug-resistant mechanisms in cancer patients.

## INTRODUCTION

Metastasis is the primary reason for cancer-related mortality. This hallmark of cancer, comprises of a sequence of processes whereby cancer cells invade the circulatory system and spread to distant organs. A growing body of evidence suggests that tumor cells spread to distant sites much earlier than was previously believed. Circulating tumor cells (CTCs), first observed by Thomas Ashworth in the blood of a metastatic cancer patient, were identical to the cells of the distant solid tumor from which they have been derived from.

### Role of CTCs in hematogenous metastasis

**Figure 1:**
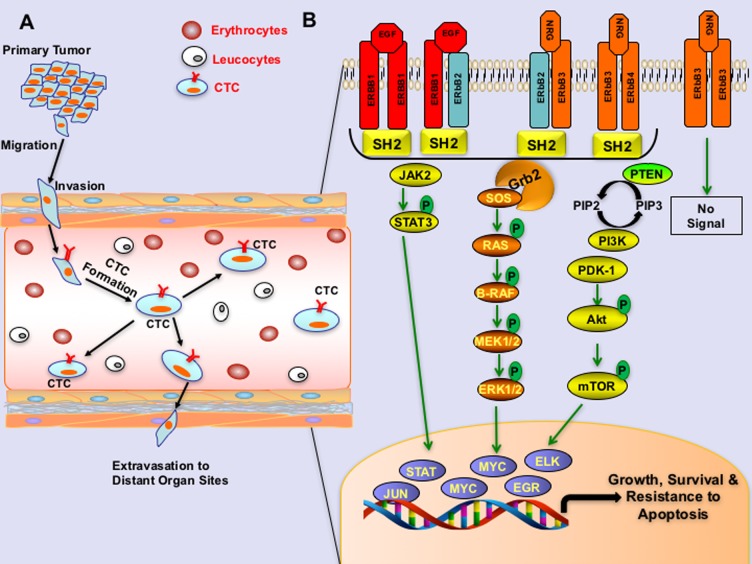
ERBB signaling in circulating tumor cells A. Schema representing the migration of circulating tumor cells into the bloodstream. B. An illustration showing ERBB signaling pathway in circulating tumor cells. ERBB receptors undergo homo or hetero dimerization, then subsequent trans or auto phosphorylation after binding to the respective ligands like epidermal growth factor (EGF), transforming growth factor - alpha (TGF-α) or neuregulin (NRG1) as indicated. ERBB2 binds no ligand with high affinity, and ERBB3 homodimers are catalytically inactive due to the lack of kinase domains. Once phosphorylated and activated, receptors initiate recruitment and binding of adaptor proteins via SH2 domains, thereby activating downstream-signaling pathways that lead to the activation of various transcription factors required for survival and resistance to apoptosis.

CTCs are tumor cells, which are detached from the primary tumor and enter into the bloodstream. CTCs do not bind to extracellular matrix (ECM) and survive in the bloodstream due to their resistance to anoikis. This provides opportunities to capture CTCs by noninvasive procedures, making this a powerful tool for detecting and tracking cancer and metastasis. Hematogenous metastasis of tumor cells comprises of several sequential steps, such as the detachment of cells from the primary tumor, intravasation into the vasculature, resistance to anoikis and extravasation into the secondary site (Figure 1A). Studies on CTCs identified that most of the CTCs do not survive over time due to anoikis, and only about 2.5% of CTCs form micrometastases, and about 0.01% of CTCs progress to macro-metastases [1]. Accumulating evidence suggests that survival of CTCs are due to multiple factors including resistance to anoikis, epithelial to mesenchymal plasticity, or stem-cell like properties [2]. Studies have shown that resistance to anoikis in CTCs is attributed, at least in part, due to the aberrations of Epidermal Growth Factor Receptor (EGFR) or other ERBB family proteins (ERBB2, ERBB3 (HER3), or ERBB4 (HER4). These receptors seem to act by triggering the PI3K/Akt pathway activation, which in turn results in the upregulation of the levels of anti-apoptotic protein BCL2 and X-linked inhibitor of apoptosis protein (XIAP), which mediate resistance to anoikis and survival of tumor cells in circulation; *see* Figure 1 [3, 4].

### ERBB signaling aberration in cancers

ERBB signaling is often dysregulated in a wide variety of solid tumors including breast, lung, ovarian and glioblastoma, where aberrant expression of ERBB receptors are found to be associated with poor clinical outcome and survival [5, 6]. Gene expression profiles and genome sequencing of CTCs identified ERBB2 (HER2) amplification, mutations in the PI3-Kinase subunit Alpha (PIK3CA) gene and in the fibroblast growth factor receptor (FGFR2) to be critical for the survival of CTCs [2, 3]. Other than the overexpression or mutations of ERBB receptors, cognate growth factors, which bind to the respective ERBB receptors, instigate autocrine signaling in CTCs. According to this theory, signaling cues are tuned by multiple factors including receptor overexpression, mutations or interactions of stimulatory ligands with their corresponding receptors for survival signaling in CTCs. Therefore, therapeutic targeting of ERBB family members, using monoclonal antibodies or small molecule inhibitors not only retard tumor growth and metastasis but also have the potential to eliminate CTCs [4].

### Unexpected roles of CTCs in Ovarian cancer and Glioblastoma

Ovarian cancer metastasizes primarily through peritoneal spreading (also known as peritoneal seeding), and Glioblastoma Multiforme (GBM) seldom metastasizes outside the central nervous system. Based on the nature and distribution of ovarian cancer and GBM, the precise contribution of CTCs to metastasis has been under-studied. Studies using parabiosis models on ovarian cancer, where two mice were surgically joined for a shared a circulatory system [7] and clinical samples of GBM [8, 9], demonstrated that CTCs were present in the blood samples collected from parabiosis model of ovarian cancer and in the patients of GBM.

### ERBB3 signaling in CTCs of high-grade serous ovarian cancer (HGSOC)

ERBB receptor family plays a key role in normal development of ovarian follicles by regulating the growth of ovarian surface epithelium [10]. Approximately 70%of HGSOC samples contain either amplification or mutations or increased expression of ERBB receptors [11]. Studies using parabiosis mouse models showed that more than 95% of CTCs collected from mice bearing ovarian tumors and 90% of CTCs from ovarian cancer patients are ERBB3 positive. Furthermore, ERBB3 levels positively correlate with total tumor burden and overall survival of ovarian cancer patients [7]. Clinical studies demonstrate completely random. Rather, the tropism of ovarian cancer cells towards the omentum is significant, and the omentumremains the preferred site for ovarian cancer metastasis [7]. However, the presence of sub-mesothelial disease within the peritoneal cavity raises the possibility of alternate routes of metastasis during ovarian cancer progression. Using a parabiosis model, we and our collaborators have demonstrated that ovarian cancer cells expressing high levels of human ERBB3 can metastasize hematogenously to the omentum [7]. Additionally, tumor cells within the blood vessels in the omentum also showed high levels of ERBB3. Furthermore, ERBB3/NRG1 signaling activates PI3K and SRC pathways, which promoted epithelial-to-mesenchymal transition and CTC formation of ovarian cancer cells [7]. One of the studies from our collaborators using microfluidics-based isolation of tumor cells by targeting ERBB3 protein from blood samples of a parabiosis model of ovarian cancer was able to capture more than 90% of CTCs in the blood samples [7]. In addition, another study reported that CTCs are present in the blood of ovarian cancer patients [12], supporting the notion that the hematogenous route is an important mode of metastasis of ovarian cancer cells. Consistent with the findings that ERBB3 promotes hematogenous metastasis of ovarian cancer, depletion of ERBB3 by siRNA or treatment with ERBB3-specific monoclonal antibodies reduced the formation of CTCs and the incidence of hematogenous metastasis of ovarian cancer in mouse models [7].

In conjunction with the studies, which demonstrate the role of ERBB3 on metastasis of ovarian cancer, another study using immunohistochemical analysis identified that high levels of ERBB3 are associated with poor outcome in ovarian cancer patients [13]. Furthermore, evaluation of tissue arrays prepared using tumors from ovarian cancer patients, found that the samples which express high levels of ERBB3, also express high levels of Met [a.k.a. MET or hepatocyte growth factor receptor (HGFR)] receptors. In complement, crosstalk of ERBB3 receptors with MET receptors leads to resistance to monoclonal antibodies or small molecule inhibitors, that target ERBB receptors [14]. Although ERBB3 lacks a kinase domain, ERBB3 dimerize with ERBB2 which activates PI3K/AKT, MEK/MAPK, and JAK/STAT signaling cascades for tumor initiation and progression [4].

### ERBB3 receptors as therapeutic agents

Several therapeutic agents are being used against the ERBB family members of ERBB2 and/or EGFR for treatment of human cancers in the clinic. In contrast, there has been relatively less emphasis on ERBB3 as a molecular target for therapy and no targeted therapies for ERBB3 have been approved for cancer treatment till to date (Table 1).

**Table 1:** List of monoclonal antibodies target ERBB receptors for cancer therapy

Name of the Antibody	Target Receptor	Antibody Type	Cancer type	Status in Therapy
Cetuximab (Erbitux)	EGFR	Monoclonal Antibody	Pancreatic Cancer, Head and Neck Cancer, Colorectal Cancer	Approved by FDA in 2004
Panitumumab (Vectibix)	EGFR	Monoclonal Antibody	Colorectal Cancer	Approved by FDA in 2006
Trastuzumab (Herceptin)	ERBB2	Monoclonal Antibody	HER2-positive breast cancer HER2-positive gastric Cancer	Approved by FDA in 1998
Pertuzumab (Perjeta)	ERBB2	Monoclonal Antibody	Breast Cancer and Ovarian Cancer	Phase-II (Breast Cancer) Phase-III in combination with chemotherapy (Ovarian Cancer)
Margetuximab (MGAH22)	ERBB2	Monoclonal Antibody	HER2-positive Breast Cancer HER2-positive Gastric Cancer	Approved by FDA in 2016
T-DM1 (Ado-trastuzumab emtansine, Kadcyla)	ERBB2	ERBB2 antibody-conjugated with anti-mitotic agent	HER2-positive breast cancer	Approved by FDA in 2013
Patritumab (AMG-888, U3-1287)	ERBB3	Monoclonal Antibody	Lung Cancer Breast Cancer Head and Neck Cancer	Phase-III (Lung Cancer) Phase-II (Breast Cancer) Phase-I (Head and Neck Cancer)
MM-121	ERBB3	Monoclonal Antibody	Breast Cancer Ovarian Cancer	Phase-II in combination with chemotherapy (Breast and Ovarian Cancers)
MM-111	ERBB2/ERBB3	Bispecific Monoclonal Antibody	Gastric Cancer, Breast Cancer and Esophageal Cancer	Phase-I (Breast) Phase-II (Gastric and Esophageal Cancer)

Since ERBB3 does not have a kinase activity, targeting ERBB3 using monoclonal antibodies is the only strategy to block ERBB3-mediated signaling. Importantly there are several monoclonal antibodies such as MM-121 and MM-111 (Merrimack Pharmaceuticals, Cambridge, MA) and U3-1287/AMG-888 (Amgen Inc., Thousand Oaks, CA) targeting ERBB3 are under preclinical and clinical development [15]. In brief, the current research suggests that ERBB3 is an emerging target in cancer. Thus, studies to capture ERBB3 expressing cells in cancers will improve our understanding of the roles of ERBB3-mediated tumor progression and metastasis.

### EGFR Signaling in CTCs of Glioblastoma Multiforme

Most brain and spinal cord tumors develop from glial cells, which are the supporting cells of the brain and tumors that arise from the glial cells are referred to as gliomas [16]. There are three types of glial cells known as astrocytes, oligodendrocytes, and ependymal cells. When the brain is injured, astrocytes form scar tissue which helps to repair the damage. The tumor arising from the astrocytic cells are known as astrocytomas or glioblastoma, also known as glioblastoma multiforme (GBM) [17].

### Glioblastoma

GBM is an aggressive form of glioma associated with a median patient survival of 12 to 14 months after any treatment modality, including surgery, radiation or chemotherapy. Given the low rate of extracranial metastases (0.4-0.5%), GBM patients have participated in organ donation programs. A study by Muller et al. demonstrated that the occurrence of CTCs in the peripheral blood is a more frequent event than overt extracranial metastases in GBM patients [8]. Importantly the fact that 20% of the GBM patients have CTCs in peripheral blood, further challenges the widespread belief of safe organ donation.

### Role of EGFR on CTC formation in Glioblastoma

Studies using clinical samples showed that EGFR amplification is associated with CTC formation in GBM, indicating that EGFR signaling is a critical factor for CTC formation and extracranial spreading of GBM [8, 9]. EGFR is amplified or mutated in approximately half of GBM patients, and 20% of GBM tumors express a mutant form of EGFR known as EGFRvIII, that can be constitutively activated without ligand activation [4]. However, the role of ERBB2 and ERBB3 signaling in disease progression and resistance to therapy remains largely unknown. *In vitro* studies showed that GBM cell lines are sensitive either to ERBB2-inhibiting antibodies or dual tyrosine kinase inhibitor lapatinib, which targets EGFR and ERBB2, suggesting that ERBB2 and EGFR have critical roles in GBM cell growth and metastasis [18]. Importantly, a study employing next-generation sequencing of 1,000 cancer-associated genes from primary lesions, pulmonary metastases, and normal central nervous system tissues, identified mutations in the EGFR, RB transcriptional corepressor 1 (RB1) and SET domain containing 2 (SETD2) genes in the metastatic site, but not in the primary tumor site [9]. The selective identification of genes in tumor cells at the metastatic site compared to primary site suggests an evolution in the mutational status, which has occurred since the migration of tumor cell from the primary site, which might be a critical determinant of the fate of CTCs. At present, the role of these new mutations on the functions of CTC is largely unknown. Therefore, comprehensive characterization of mutations in primary tumors, metastatic tumors, and CTCs are required for a deeper understanding of the roles of acquired mutations in tumor evolution and metastasis.

## CONCLUDING REMARKS AND FUTURE DIRECTIONS

Most of the cancer deaths are due to metastasis rather than primary tumors. Ovarian cancer is the most frequent cause of deaths among gynecological cancers [7, 19, 20]. Similarly, GBM is one of the aggressive forms of malignancy, which arises within the brain and infiltrates to other parts of the brain rapidly. Therefore, capturing CTCs in these cancers will provide unprecedented opportunities for a real-time sampling of tumors, to monitor their response to therapy and drug resistance mechanisms. Considerable research has been conducted to characterize CTCs. However the precise mechanisms that regulate the development of CTCs, dormancy, and their role in the formation of micro-metastasis are not fully understood.

CTCs could be employed as a source of readily accessible genetic information of patient's tumors. Therefore, the potential use of CTCs in future personalized medicine strategies for cancer therapy such as tailoring therapy based on molecular characterization of CTCs in cancer patients will have clinical implications. However, better methods of isolating CTCs in large quantities are required for characterization to further exploit the utilities of CTCs in the clinic.

The prevailing model of metastasis suggests that metastatic subpopulation of cancer cells display characteristics of cancer stem cell-like (CSCs) traits and exhibits features of epithelial-to-mesenchymal transition (EMT). However, EMT and CSC paradigms have been highly challenged in the last several years, because the pathological evidence of EMT or CSCs in human cancer samples has not been well establishedyet. Furthermore, tumor cells also undergo the process of mesenchymal-to-epithelial transition (MET), which is the reverse of EMT and tumor cells may lose or alter expression of CSC markers during metastasis [21]. Thus, the use of EMT or CSC markers alone would not be adequate to isolate CTCs, that represent primary tumors. Current research reveals that ERBB family members initiate intracellular pathways that support the formation and survival of CTCs derived from GBM and ovarian cancer. Therefore, targeting ERBB receptor family members like EGFR, ERBB2 or ERBB3 alone or in combination with other epithelial markers such as EpCAM, will provide opportunities to detect and isolate CTCs that represent primary tumors of ovarian and glioblastoma.

BOX 1 **ERBB signaling mechanisms in CTCs**

The ERBB family comprises of four receptors such as EGFR, ERBB2, ERBB3, ERBB4 and 13 polypeptide ligands contains a conserved epidermal growth factor (EGF) domain. Each receptor contains an extracellular ligand-binding domain, a transmembrane domain, and an intracellular tyrosine kinase domain. ERBB receptors normally exist as inactive monomers, with a molecular structure preventing dimerization, except ERBB2. Homo-or heterodimer formation between ERBB family members occurs upon ligand binding, where ERBB2 do not contain a ligand binding domain. However, ERBB2 act as the key receptor of ERBB family receptors due to their dimerizing capacities either with EGFR, upon EGF binding, or with ERBB3, upon NRG1 binding. Ligand binding further leads to trans-phosphorylation and tyrosine kinase activation of receptors, except ERBB3 receptor, which do not have a catalytically active kinase domain.

Independent of any ligand binding, overexpression of ERBB2 also leads to homo-dimerization of ERBB2. Dimerization ERBB receptors can instigate a number of potent signaling pathways, including the mitogen-activated protein kinase pathway and the PI3-kinase (PI3K)–AKT pathways [4]. Other than the PI3K-AKT pathway, ERBB signaling also relies on PI3K–AKT, ERK/MAPK, PLCγ1/PKC and STAT pathways for evasion of apoptosis and survival of tumor cells; see Figure 1 [4].
